# Preservation of vision by transpalpebral electrical stimulation in mice with inherited retinal degeneration

**DOI:** 10.3389/fcell.2024.1412909

**Published:** 2024-08-14

**Authors:** Kasim Gunes, Karen Chang, Anton Lennikov, Wai Lydia Tai, Julie Chen, Farris ElZaridi, Kin-Sang Cho, Tor Paaske Utheim, Chen Dong Feng

**Affiliations:** ^1^ Department of Ophthalmology, Schepens Eye Research Institute of Mass Eye and Ear, Harvard Medical School, Boston, MA, United States; ^2^ Department of Histology and Embryology, School of Medicine, Marmara University, Istanbul, Türkiye; ^3^ Department of Medical Biochemistry, Oslo University Hospital, Oslo, Norway; ^4^ Department of Ophthalmology, Oslo University Hospital, Oslo, Norway

**Keywords:** electrical stimulation, retinitis pigmentosa, photoreceptor degeneration, retina, cones, rods

## Abstract

**Introduction:**

The potential neuroprotective and regenerative properties of electrical stimulation (ES) were studied in rhodopsin knockout mice (*Rho* ^*−/−*^), a murine model of inherited retinal degeneration. The study focused on assessing the impact of varying ES frequencies on visual functions and photoreceptor cell survival in *Rho* ^*−/−*^ mice.

**Methods:**

To elucidate the impact of electrical stimulation on cone survival, *Rho* ^*−/−*^ mice received either sham or transpalpebral ES using biphasic ramp or rectangular waveforms at 100 µA amplitude, starting at six weeks of age. The treatment duration spanned from one to three weeks. The optimal treatment frequency of ES sessions was determined by applying ES every one, two, or three days in three separate groups of *Rho* ^*−/−*^ mice. The sham group received daily treatments without the application of ES.

**Results:**

Our study revealed significant improvement of visual function in *Rho* ^*−/−*^ mice following daily or every-other-day noninvasive transpalpebral ES, as evidenced by electroretinogram and optomotor response-based visual behavior assays. Concurrently, assessment of outer nuclear thickness and immunohistochemistry for the cone photoreceptor cell marker PNA demonstrated pronounced increases in the survival of rods and cones and improvement in the morphology of the inner and outer segments.

**Discussion:**

This study underscores the protective effect of non-invasive ES in rhodopsin knockout-induced retinal degenerative disorders, providing a foundation for developing targeted therapeutic interventions for retinitis pigmentosa.

## Introduction

Retinitis pigmentosa (RP) is a group of hereditary retinal degenerative conditions characterized by continuous photoreceptor loss. Photoreceptors are light sensing cells that transform light energy into electrical signals of neurons. Loss of photoreceptors leads to visual impairment and adaptive responses of retinal remodeling (Marc et al., 2003; Jones et al., 2016; Rodriguez Villanueva et al., 2020). The RP symptoms begin with night vision loss, progress to decreased visual sharpness, and eventually leads to a restricted visual field. By age 40, most individuals with RP are considered legally blind (Hamel, 2006; Hartong et al., 2006; Jones et al., 2016; Amamoto et al., 2022). As the disease advances, patients struggle more with daily tasks and lose independence. This decline in quality of life places a heavy burden not only on the patients but also on their families, caregivers, and society at large. With only one approved gene therapy for RP patients with RPE65 gene mutation, which represents 2% cases of recessive RP and approximately 16% of leber congenital amaurosis (Morimura et al., 1998; Ferrari et al., 2011), the majority are left with supportive care and limited options to significantly improve or prevent RP related vision loss, representing a significant unmet clinical need (O'Neal and Luther, 2024).

Transpalpebral Electrical Stimulation (TpES) has gained increasing attention for its potential to improve vision in patients with retinal diseases including age-related macular degeneration (AMD) and RP (Anastassiou et al., 2013; O'Clock and Jarding, 2009; Shinoda et al., 2008). This method involves delivering electrical micro-currents through the skin of the eyelid, directly targeting the retina to modulate its cellular functions (Morimoto et al., 2002; Chang et al., 2021; Enayati et al., 2024; Li et al., 2024). The increasing popularity of TpES can be attributed to several factors: its non-invasive nature reduces patient discomfort and the need for surgical procedures, its cost-effectiveness compared to many other treatments and favorable safety profile, making it a reliable choice for long-term use (Yang et al., 2022; Enayati et al., 2024). While previous studies have demonstrated the potential benefits of noninvasive ES treatment (Enayati et al., 2020; Chang et al., 2021), the optimal ES parameters; waveforms and treatment frequencies are still unclear, resulting in inconsistent clinical outcomes. To address this knowledge gap, the present study compares the efficacy of rectangular and ramp waveforms of ES, as well as treatment frequencies, in preventing photoreceptor cell loss in rhodopsin deficient (*Rho* ^−/−^) mice.

## Methods

### Animals

*Rho* ^*−/−*^ mice were originally generated at Trinity College in Dublin, Ireland (Humphries et al., 1997) A colony of *Rho* ^−/−^ mice, was maintained at the Animal Facility of the Schepens Eye Research Institute of Mass Eye and Ear with access to food and water *ad libidum*. Over the course of 3 months after birth, *Rho* ^−/−^ mice gradually lose their photoreceptors and electroretinography (ERG) responses (Humphries et al., 1997). *C57BL6* mice were purchased from Jaxon Labs and used as control. All animals were housed in a 12-h light/dark cycle at specific-pathogen-free (SPF) animal facility at Schepes Eye Research Institute. The facility is accredited by American Association for Laboratory Animal Science. All animal experiments were approved by the Institutional Animal Care and Use Committee of the Schepens Eye Research Institute and were conducted in compliance with the guidelines of the ARVO (Association for Research in Vision and Ophthalmology, Rockville, MD, United States of America). Genotyping for the *Rho* ^*−/−*^ mice was carried out using Transnetyx’s outsourced PCR genotyping services (www.transnetyx.com) via a real-time PCR assay. At the conclusion of the experiment, mice were euthanized through CO2 inhalation in a custom-built plexiglass chamber. The CO2 fill rate was regulated to be between 30%–70% of the chamber volume per minute, following the NIH ARAC Guidelines for Euthanasia of Rodents Using Carbon Dioxide (Shomer et al., 2020).

### Noninvasive electrical stimulation

Under isoflurane anesthesia, conducting electrode gel (Spectral 360; Parker Laboratories, Fairfield, NJ, United States of America) was placed on the mouse’s upper and lower eyelids to provide optimal contact and conductivity between the electrodes and the skin. Mice were stimulated by a STG4000 pulse generator (Multi Channel Systems, Reutlingen, Germany). To investigate the possible neuroprotective effects of noninvasive ES on mouse photoreceptors, the portable cathode probe was applied for 1 minute at each of four locations on the skin surrounding the mouse orbit: two on the upper and two on the lower eyelids. ES was produced as a sequence of biphasic rectangular (100 μA, 20 Hz) or biphasic ramp (100 μA, 20 Hz) pulse series. The mouse’s abdomen was connected to the anode electrode. Mice were randomized into groups that received ES in either the left or right eye. In the sham group, the probe was applied to the four locations on the eyelid for 4 min (1 minute at each location) in anesthetized mice without engaging the current in pulse generator. As previously reported, we did not observe significant differences in ERG b-wave amplitudes (Yu et al., 2020) and VA and CS values (Xiao et al., 2019) between sham and untreated eyes of *Rho* ^*−/−*^ mice, at least when examined at 6 to 9 weeks of age.

### Electroretinography

Photopic electroretinograms (ERGs) were recorded once a week, starting a day before the first ES to establish the baseline. The ERG was performed as previously reported (Yu et al., 2020). The mice were given an intraperitoneal injection of Ketamine (100 mg/kg; Dechra Vet Products, Overland Park, KS, United States of America, 383017-01) and Xylazine (20 mg/kg; Covetrus North America, Dublin, OH, United States of America, 1XYL006). Tropicamide 0.5% (Sandoz, West Princeton, NJ, United States of America) was used to dilate the pupils. During the recording, mice were placed on a 37°C warming pad in a Ganzfeld bowl (Diagnosys LLC, Lowell, MA, United States of America). Two contact electrodes were positioned centrally on the corneas of each eye to record electroretinographs. We used GenTeal gel (Novartis, Basel, Switzerland) to lubricate the electrodes for optimal contact. Ground and reference electrodes were placed subcutaneously near the base of the tail and the top of the forehead, respectively. The ERG b-wave amplitude values were normalized to the baseline reading recorded at 6 weeks of age before the initial ES session of each mouse and presented as b-wave amplitude relative to the baseline values.

### Optomotor response

As previously reported, a custom-built optomotor response (OMR) device was used to assess visual acuity (VA) and contrast sensitivity in *Rho* ^*−/−*^ mice following ES/sham treatments (Shi et al., 2018; Pan et al., 2023). Mice were placed on a stand set surrounded by four 15.6-inch LCD monitors (Acer 16PM6Q, Acer, Schaumburg, IL, United States of America), and head movement was observed when presenting the mouse images of moving black and white bars. Bar width and brightness were altered increasingly until an OMR response was observed using a custom-built OMR device (Shi et al., 2018). The staircase paradigm was used for spatial frequency and sinusoidal gratings with black and white stripes to measure VA and CS. OMR was performed on 6-week-old mice and then observed weekly (for 3 weeks after the beginning of TpES) to assess the mouse’s visual function. Scoring of mouse head movement in response to visual stimuli was performed by two observers that were masked to the treatment groups; an agreement on a positive or negative OMR response must be mutually reached at the same time, or otherwise, the observation was considered false. All data collection and analyses were performed in a masked fashion. The VA and CS data were normalized to the baseline OMR reading recorded at 6 weeks of age before the initial ES session of each mouse and was represented as a relative fold change of the baseline.

### Immunohistochemistry and quantification for cone survival

The eyes were enucleated and fixed in 4% paraformaldehyde for 2 h, cornea, lens, and iris were removed. Resulting eye cups were cryoprotected with 20% sucrose for 20 min at room temperature, embedded in an optimum cutting temperature media (Tissue-Tek OCT Compound; Sakura, Torrance, CA, United States of America), and cryosection sagittally at 18 μm thickness. Retinal sections were cut in the superior-inferior or nasal-temporal axis through the optic nerve head. Eyecup sections were blocked for 30 min at 37°C with a blocking buffer made of 1% BSA (Sigma-Aldrich, St. Louis, MO, United States of America), 0.1% Triton X-100 (Millipore Sigma), and 0.1% Tween20 (Sigma-Aldrich, St. Louis, MO, United States of America) in phosphate-buffered saline (PBS). Cone outer segments (COS) were identified by Alexa488-conjugated peanut agglutinin (PNA) labeling (1:200; L21409, Life Technologies, Carlsbad, CA, United States of America) in a blocking solution at 4°C overnight (Blanks and Johnson, 1983; Hageman and Johnson, 1986). DAPI (4′,6-diamidino-2-phenylindole, 62,247, Thermo Fisher Scientific, Fisher Scientific, Waltham, MA, United States of America) counterstain was used to visualize cell nuclei, and slides were mounted with DAKO fluorescent mounting solution (DAKO, S3023, Carpinteria, CA, United States of America). Images were captured using a Leica SP8 confocal microscope (Leica Microsystems, Wetzlar, Germany). The number of rows of DAPI-positive nuclei in the outer nuclei layer (ONL) was counted on cross-section images obtained from the superior and inferior mid-central retina of each eye, and the results were averaged per animal. ONL thickness was measured by ImageJ (National Institute of Health, Bethesda, MA, United States of America). PNA-positive cones’ inner and outer segments were counted, and the percentage of PNA-positive cones was calculated. Each animal’s average is calculated by adding all the data from four sections, each including four images—two from the superior and two from the inferior retina per eye.

### Statistical analysis

The statistical analysis in this study was conducted using GraphPad Prism (https://www.graphpad.com/scientific-software/prism/). Data were tested for normality and were reported as mean ± standard error of the mean (SEM) for each group. To compare two individual groups, the Student’s t-test was employed. For comparisons between multiple groups, a one-way analysis of variance (ANOVA) followed by Tukey’s multiple comparisons test was used. For repeated observations in the same animals (Figure 5; Supplementary Figure S4) Holm-Šídák test with multiple comparisons correction was used. Statistical significance was defined as a P value less than 0.05. In the figures, statistical significance is indicated as follows: not significant (n.s.) for *P* > 0.05, * for *P* < 0.05, ** for *P* < 0.01, and *** for *P* < 0.001.

## Results

### TpES at a ramp waveform is optimal for maintaining visual function in *Rho* ^*−/−*^ mice

**FIGURE 1:**
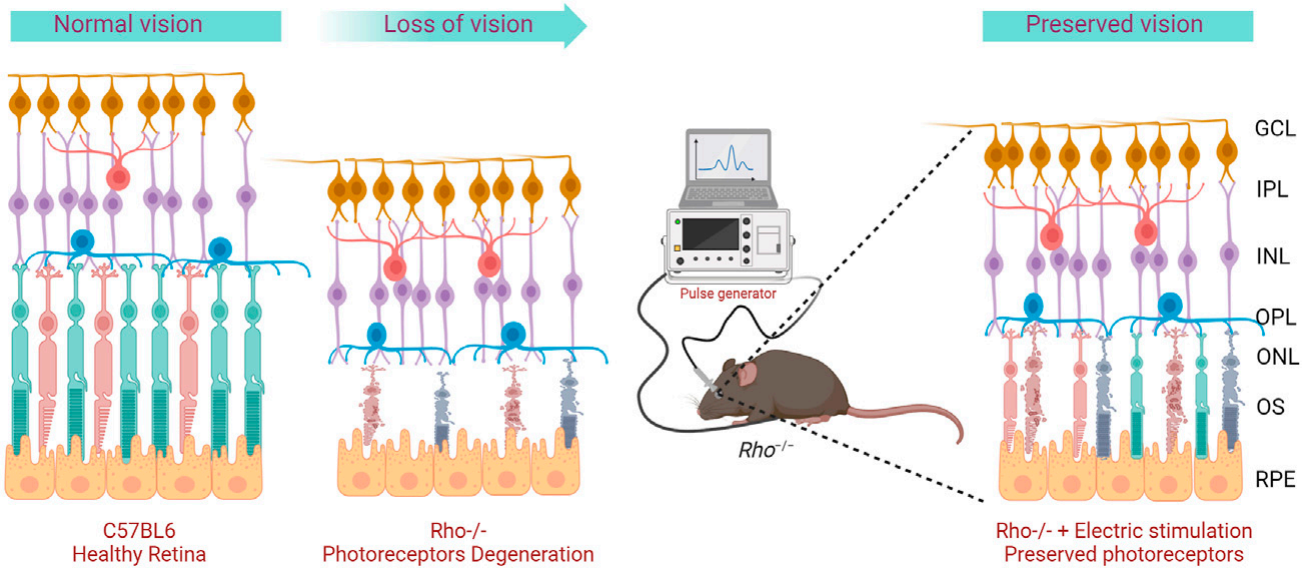
Graphical representation of the project concept. *Rho* ^*−/−*^ mice display progressive photoreceptor degeneration and loss of visual functions over time. Repeated transpalpebral electric stimulation delay degeneration and preserves photoreceptors.

**FIGURE 2:**
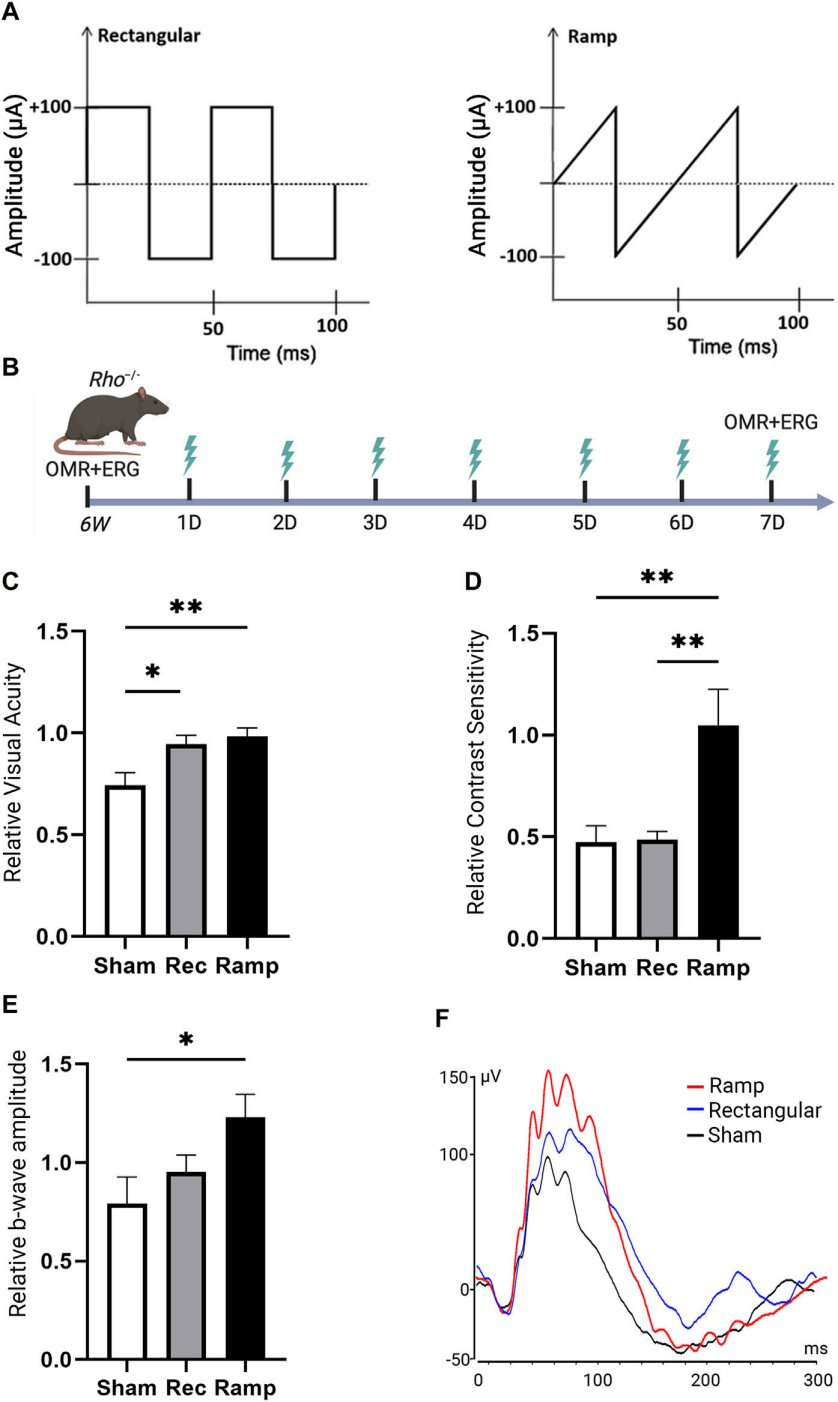
Transpalpebral electrical stimulation with the Rectangular and Ramp waveforms improves vision in *Rho* ^*−/−*^ mice. Graphical representation of rectangular and ramp ES waveforms used in the study **(A)**. Summary of the experimental design and timeline **(B)**. Visual acuity VA; **(C)** and Contrast Sensitivity CS; **(D)** of the rectangular (Rec) or ramp (Ramp) ES-treated eyes as assessed by OMR in 7 weeks old *Rho* ^*−/−*^ 7 days after the first ES or in the sham (Sham) group. Data are presented as VA or CS values relative to their baseline levels acquired before the initial stimulation at 6 weeks and established as 1. Photopic 600 ERG recordings from *Rho* ^*−/−*^ mice showing quantification of b-wave amplitudes and **(E)** representative ERG plot taken 7 days of ES or Sham treatment **(F)**. Statistical significance was evaluated using one-way analysis of variance (ANOVA), with *p*-values <0.05 deemed significant. For all statistical data, an asterisk indicates *P < 0.05, **P < 0.01, and values are reported as mean ± SEM. (VA, CS n = 7 mice/group; ERG n = 6 mice/group).

We studied the therapeutic effects of noninvasive TpES in an animal model of inherited photoreceptor degeneration. *Rho* ^*−/−*^ mice, received ramp or rectangular TpES in one eye for 7 days starting at 6 weeks of age (Figure 1). The baseline OMR was recorded before first TpES session that was administered 4 min per day, daily using biphasic rectangular (100 μA, 20 Hz) or biphasic ramp (100 μA, 20 Hz) pulse series (Figure 2A) following our previously established ES paradigm (Yu et al., 2020). Sham stimulated mice were used as control. The graphical summary of the experimental timeline is present in Figure 2B. The OMR results indicated significant improvement of visual acuity (*P* < 0.01; Figure 2C) and contrast sensitivity (*P* < 0.01, Figure 2D) in mice stimulated with ramp waveform. The rectangular waveform stimulation has achieved improvement in visual acuity (*P* < 0.05), but not contrast sensitivity (*P* > 0.05). Only the mouse eyes that received TpES treatment at a ramp waveform demonstrated significantly improved photopic ERG wave amplitude (Figures 2E, F), when compared to the sham group.

Remarkably, we also observed significant improvements in VA (*P* < 0.01) and CS (*P* < 0.01) in the mouse eyes contralateral to the ramp treatment in *Rho* ^*−/−*^ mice (Supplementary Figure S1A). These benefits found in the contralateral eyes were not observed in rectangular waveform ES-treated *Rho* ^*−/−*^ mice (*P* > 0.05). No significant improvement (*P* > 0.05) in ERG b-wave amplitudes was detected in the eyes contralateral to ES-treatment compared to the control group (Supplementary Figure S1C). The data suggest that the ramp waveform was superior compared to the rectangular waveform in preventing photoreceptor function loss in *Rho* ^*−/−*^ mice. The observation in contralateral eyes indicates that ES especially with ramp waveform exerts an effect on the contralateral eye during the stimulation.

### Ramp ES improves cone survival and morphology in 9 weeks old *Rho* ^*−/−*^ mice

**FIGURE 3:**
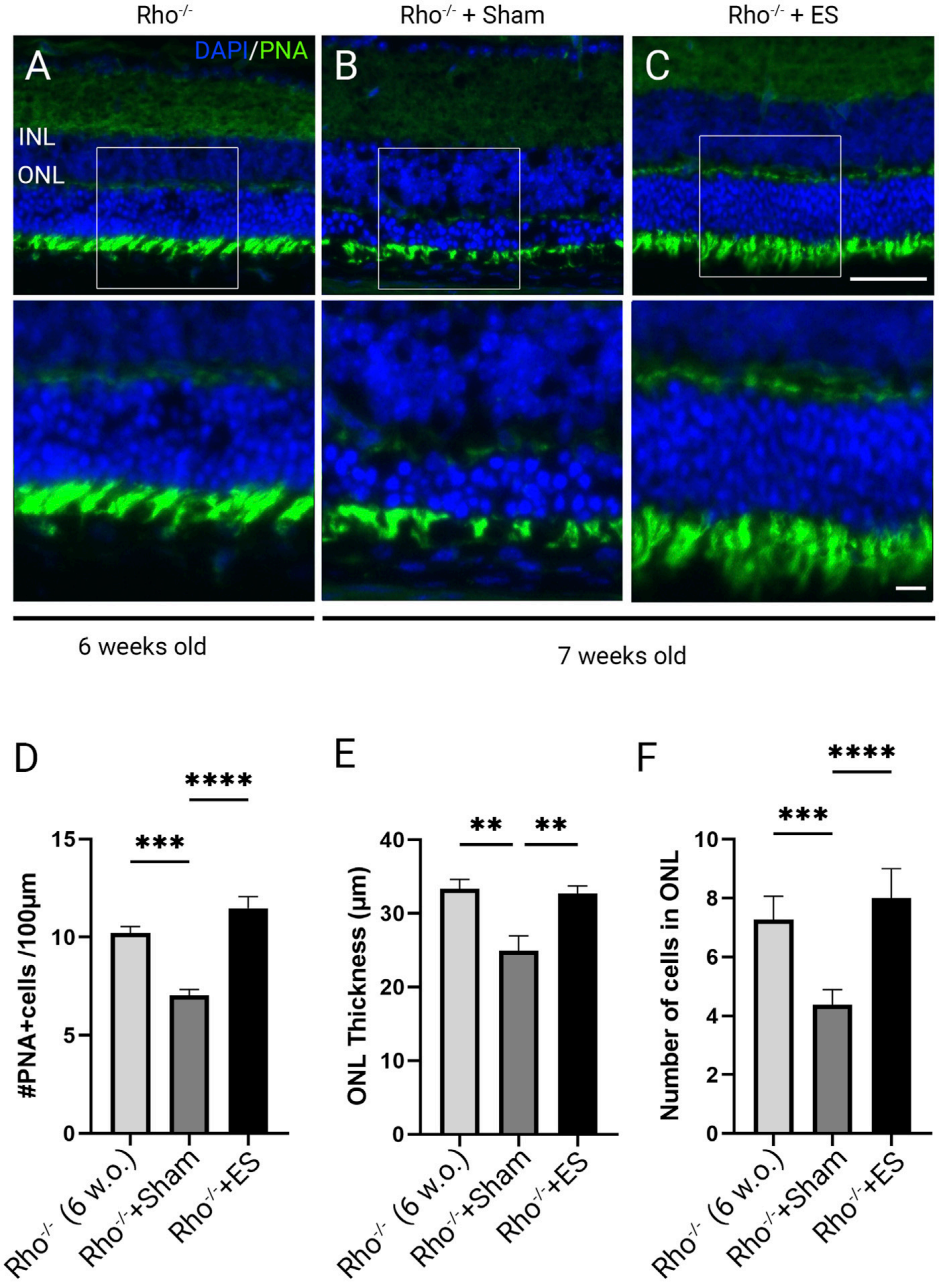
Photoreceptor degeneration in Rho^−/−^ mice and transpalpebral electrical stimulation with a ramp waveform improves photoreceptor survival. Representative morphologies of retinal sections immunolabeled with Peanut agglutinin (PNA) and counter-stained with 4′,6-diamidino-2-phenylindole (DAPI) in 6-weeks-old Rho^−/−^ mice **(A)**; 7-weeks-old Rho^−/−^ mice after receiving 1 week of Sham **(B)** or ramp ES treatments **(C)** starting at 6 weeks of age. ONL: outer nuclear layer; INL: inner nuclear layer. Scale bar = 50 μm; insert = 20 μm. Quantification of PNA-positive cells across retinal sections **(D)**. Measurement of retinal ONL thickness **(E)** and ONL cell layer qualifications of the retinal sections **(F)**. Statistical significance was evaluated using one-way ANOVA. *P < 0.05, **P < 0.01 ***P < 0.001, and values reported as mean ± SEM (C5BL6 n = 6 mice/group; *Rho* ^*−/−*^ mice n = 8 mice/group).

To investigate morphological changes and the impact of ES in retinal degeneration we performed immunolabeling using Peanut agglutinin (PNA) in retinal sections of *Rho* ^*−/−*^ and C57BL/6J wild-type control mice. PNA binding is specific to the inner and outer segments and the synaptic pedicles of cone photoreceptors (Blanks and Johnson, 1984). PNA immunolabeling demonstrated healthy photoreceptors in 6 weeks old C57BL/6J mice (Supplementary Figure S2A). Marked photoreceptor degeneration was observed in 6 weeks old *Rho* ^*−/−*^ mice (Figure 3A; Supplementary Figure S2B) with abnormal photoreceptor outer segment morphology and significantly reduced photoreceptor density (Supplementary Figure S2C; *P* < 0.001), thickness (Supplementary Figure S2D; *P* < 0.001) and ONL cell layers (Supplementary Figure S2E; *P* < 0.001). The photoreceptor degenerative progressed from 6 to 7 weeks postnatal in *Rho* ^*−/−*^ mice treated with sham stimulation, as shown by ONL thinning and loss of PNA-stained outer segment (Figure 3B). TpES-treated 7 weeks old *Rho* ^*−/−*^ mice showed significantly increased PNA + cells and thicker ONL compared to sham-treated *Rho* ^*−/−*^ mice (Figures 3C–F). The results support the neuroprotective effects of TpES on photoreceptors in *Rho* ^*−/−*^ mice.

### Daily or every other day ES treatment is required to maintain the visual benefits in *Rho* ^*−/−*^ mice

**FIGURE 4:**
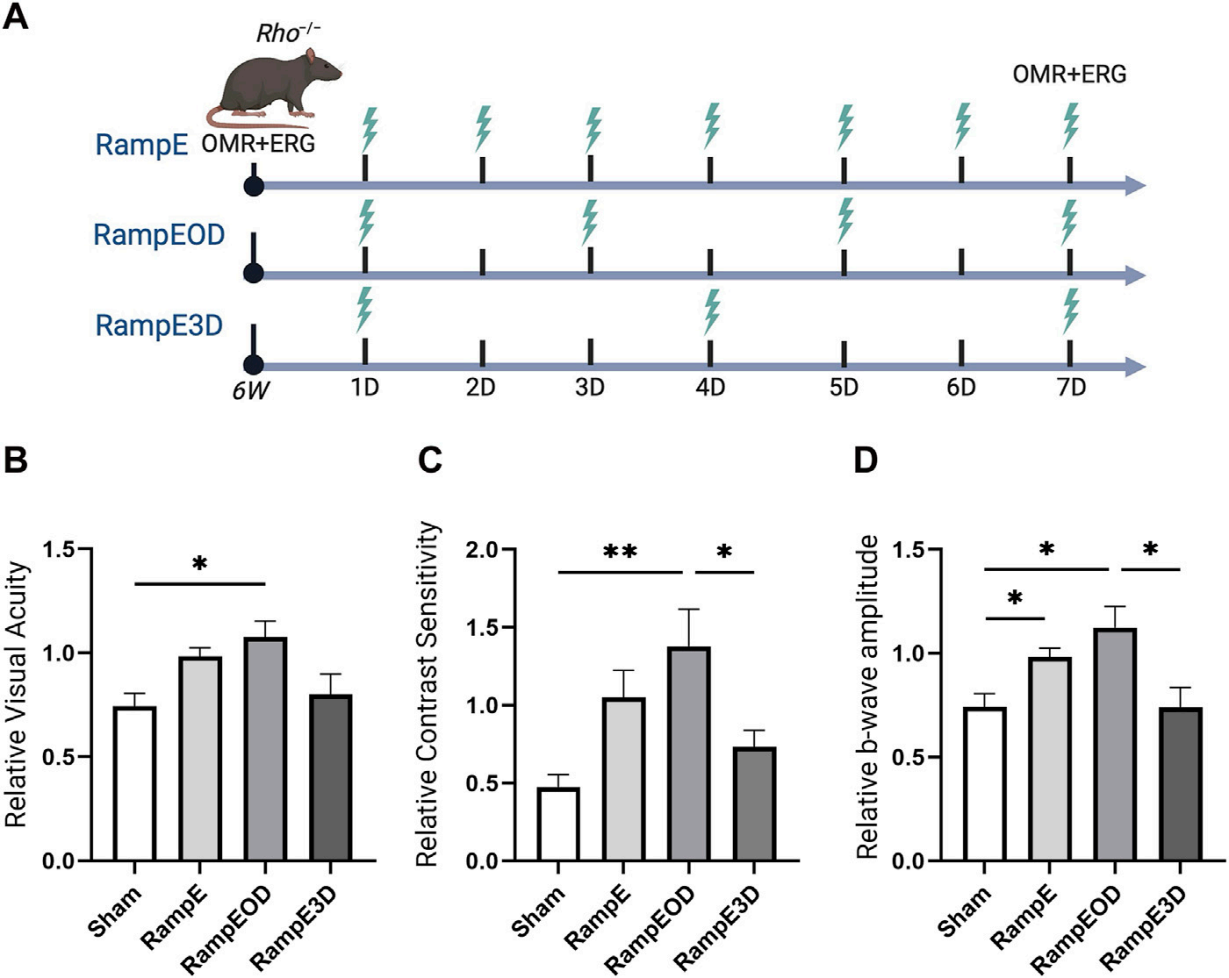
Every other days TpES schedule with Ramp waveform improves vision in *Rho* ^*−/−*^ mice. Graphical summary of the experimental design and ES timeline: RampE – Daily TpES; RampEOD – TpES every other day; RampE3D – TpES every 3 days, and Sham stimulation as control **(A)**. Visual acuity VA; **(B)** and Contrast Sensitivity CS; **(C)** of the experimental animals as assessed by OMR in 7 weeks old *Rho* ^*−/−*^ 7 days after the first ES or in sham stimulation. Data are presented as VA or CS values relative to their baseline levels acquired before the initial stimulation at 6 weeks and established as 1. Photopic 600 ERG recordings of b-wave amplitudes from *Rho* ^*−/−*^ mice treated with unliteral sham or TpES **(D)**. Statistical significance was evaluated using one-way ANOVA. *P < 0.05, **P < 0.01, and values are reported as mean ± SEM (n = 7 mice/group).

Next, we studied the optimal treatment schedule of ES using a ramp waveform in *Rho* ^*−/−*^ mice. *Rho* ^*−/−*^ mice at 6 weeks of age were subjected to ramp-TpES either every day, every other day, or once every 3 days for 1 week (Figure 4A). OMR was performed before the first ES session to establish the baseline value and at 7 days after the first TpES. We found that the eyes that received ramp waveform TpES every day performed markedly better in OMR tests (Figures 4B, C), and daily stimulations resulted in significant improvement in ERG b-wave amplitude (Figure 4D; *P* < 0.05). The ES every other day demonstrated significantly improved VA (Figure 4B; *P* < 0.05), CS (Figure 4C; *P* < 0.01), and photopic ERG b-wave amplitudes (Figure 4D; *P* < 0.05) when compared to sham-treated *Rho* ^*−/−*^ mice and mice that received ES once every 3 days. There were no significant differences in the VA, CS, and photopic ERG b-wave amplitudes between the eyes that received sham and every 3-day ES treatment (*P* > 0.05).

Marked improvements in VA were also observed in eyes contralateral to the ES treatment in mice stimulated every day and every other day, although only every other day stimulated group reached statistical significance in VA assessment (Supplementary Figure S3A; *P* < 0.05). Significant improvements in CS of the eyes are contralateral to the treatment were detected in both the every day and every-other-day ES groups (Supplementary Figure S3B; *P* < 0.01). Nonetheless, no significant improvement in ERG b-wave amplitudes was noted (Supplementary Figure S3C; *P* > 0.05). These results suggest that TpES at a treatment schedule of every other day presents an optimal therapeutic benefit with mild therapeutic benefit in the contralateral eye, implicating propagation of the electric field to both eyes during mono-ocular stimulation.

### Benefits of long-term ES application

**FIGURE 5:**
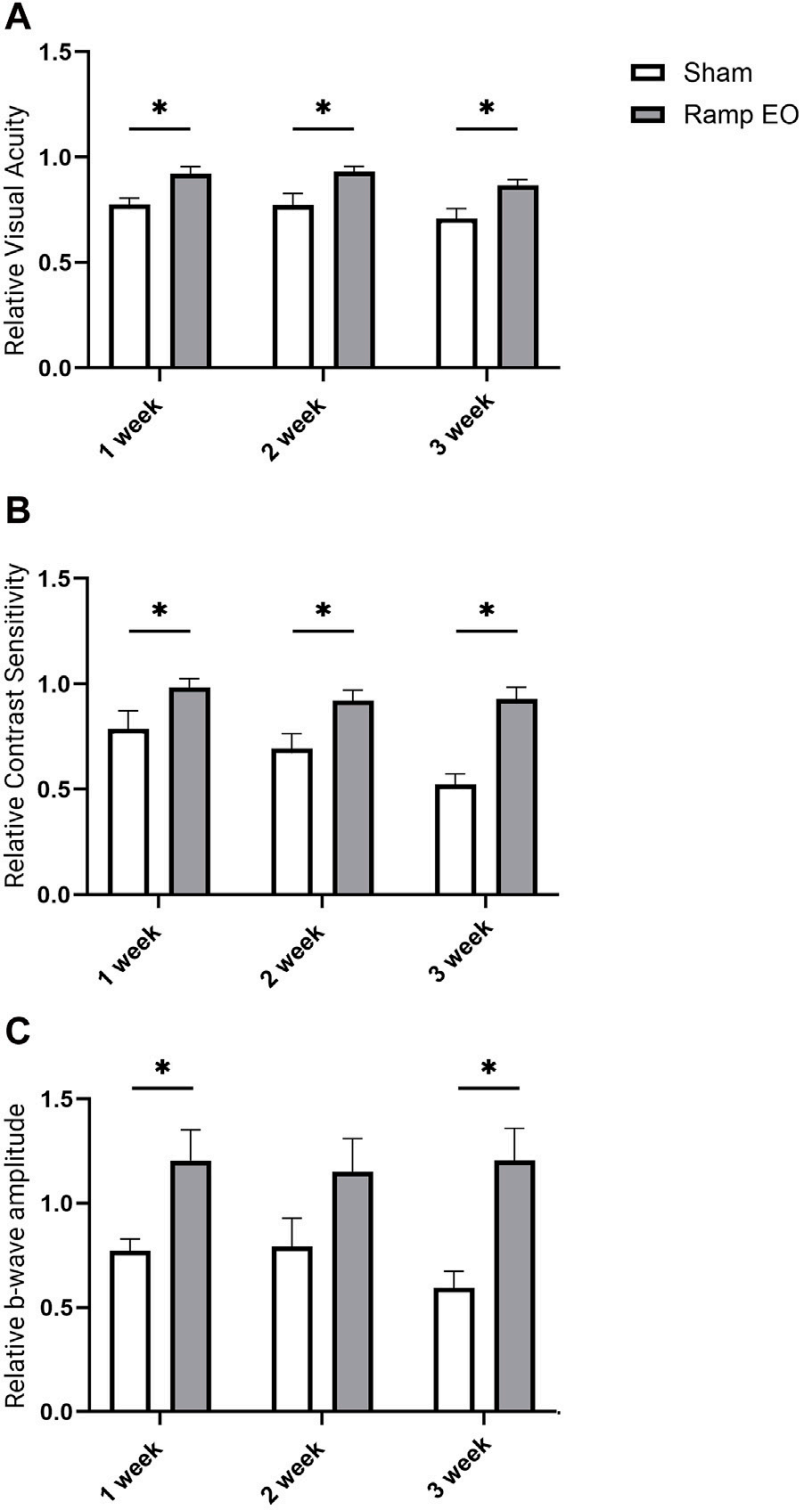
Long term transpalpebral electrical stimulation with the ramp waveform prevents vision loss in *Rho* ^*−/−*^ mice. Visual acuity VA; **(A)**; Contrast Sensitivity CS; **(B)** in *Rho* ^*−/−*^ mice sham or ramp TpES-treated every other day eyes for 3 weeks. VA and CS data are presented as values normalized to baseline values acquired at 6 weeks of age before the first stimulation and established as 1. **(C)** B-wave amplitudes of photopic 600 ERG in 3 weeks stimulation in sham or TpES-treated *Rho* ^*−/−*^ mice. Statistical significance was evaluated using Holm-Šídák test with multiple comparisons correction. *P < 0.05, **P < 0.01, or ***P < 0.001, ****P < 0.0001, and values are reported as mean ± SEM. (n = 8 mice/group).

To address the question of whether the beneficial effects of ES results in temporary improvement or can be sustained over a longer period, we performed a longer-term ES experiment. Six-week-old *Rho* ^*−/−*^ mice were treated with ramp TpES every other day for up to 3 weeks. The VA and CS were assessed before ES and every week after the first ES treatment for 3 weeks and ERG responses were recorded at the study endpoint. As expected, sham-treated *Rho* ^*−/−*^ mice exhibited significantly decreased VA, CS, and photopic ERG b-wave amplitude with progressive linear decrease. In contrast, every-other-day ES-treated eyes maintained the VA (Figure 5A; *P* < 0.05) and CS (Figure 5B; *P* < 0.05) and ERG amplitude to the baseline level without apparent declines up to 3 weeks (Figure 5C). The findings highlight the functional advantages of TpES at a ramp waveform when treated every other day in an RP model suggesting long term therapeutic benefits with continued stimulation.

Assessments of the VA, CS, and photopic ERG in the contralateral eye supported the prolonged benefits of TpES in *Rho* ^*−/−*^ mice compared to the sham-treated eyes of control mice. With the significant improvement of VA (Supplementary Figure S4A; *P* < 0.01), CS (Supplementary Figure S4B; *P* < 0.05; *P* < 0.01), and ERG b-wave amplitudes by 3 weeks of stimulation (Supplementary Figure S4C; *P* < 0.01). These findings indicate that electric field propagation into the contralateral eye and moderate beneficial effects in longer-term mono-ocular stimulation.

### Ramp ES improves cone survival and morphology in 9 weeks old *Rho* ^*−/−*^ mice

**FIGURE 6:**
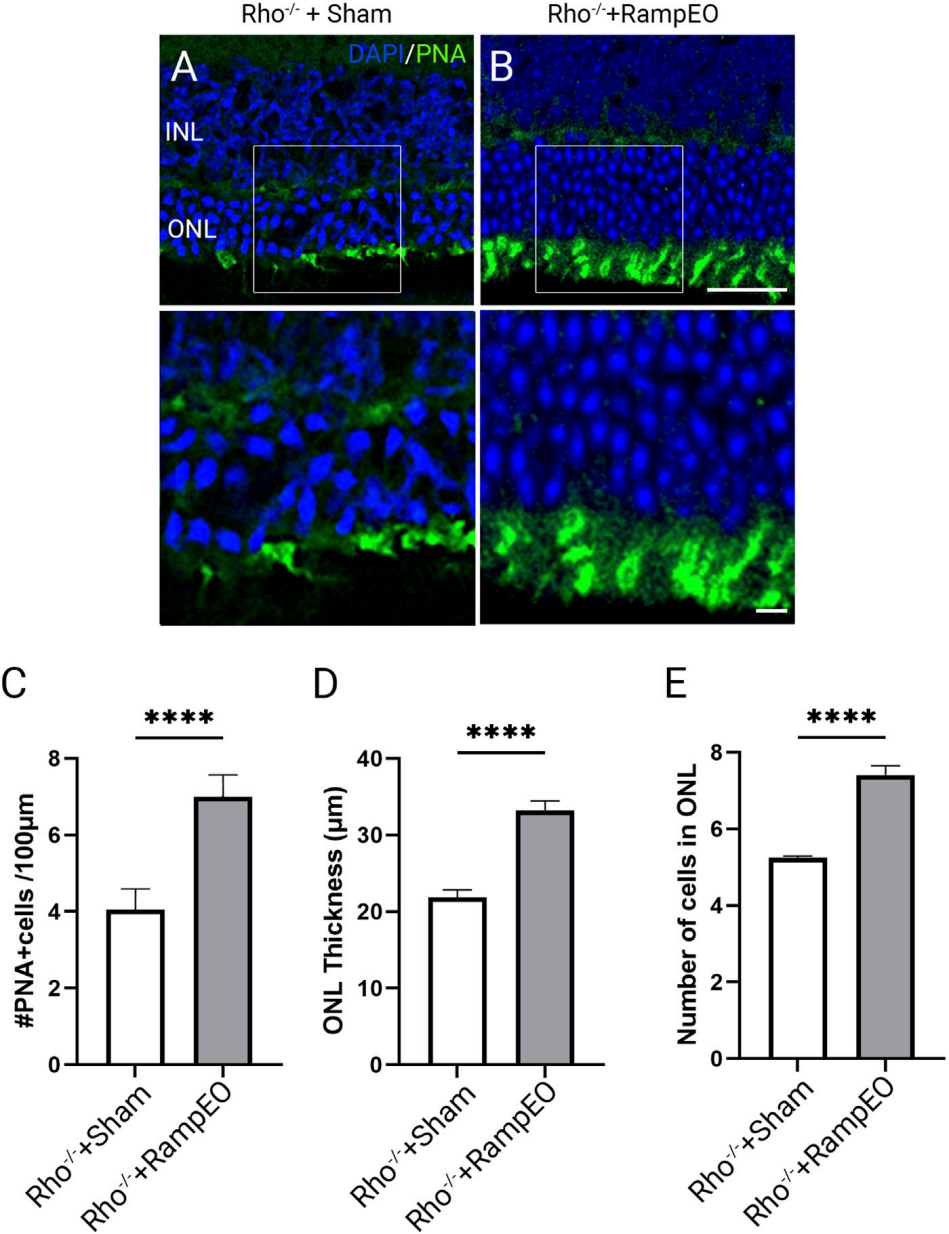
Long-term transpalpebral electrical stimulation with a ramp waveform improves photoreceptor survival in Rho^−/−^ mice. Representative images of retinal sections immunolabeled for Peanut agglutinin (PNA) and counter-stained with 4′,6-diamidino-2-phenylindole (DAPI) in 9-weeks-old Rho^−/−^ mice after receiving 3 weeks of Sham **(A)** or ramp TpES treatment every other day starting at 6 weeks of age **(B)**. ONL: outer nuclear layer; INL: inner nuclear layer. Scale bar = 50 μm; insert = 20 μm. Quantification of PNA-positive cells in retinal sections **(C)**. Measurement of retinal ONL thickness **(D)** and ONL cell layers **(E)** in TpES and sham-treated eyes. Statistical significance of the results was evaluated by unpaired t-test, with *P < 0.05, **P < 0.01 ***P < 0.001, and values reported as mean ± SEM (Sham n = 5 mice/group; Ramp EO n = 6).

We next investigated the morphology in 9-week-old *Rho* ^*−/−*^ mice subjected to sham and ramp TpES every other day. In 9-week-old *Rho* ^*−/−*^ mice that received sham stimulation, drastic loss of ONL thickness and PNA-labeled outer segment integrity were observed (Figure 6A). The TpES-treated eyes demonstrated thicker ONL and better preserved PNA-labeled outer segment (Figure 6B). There were significant increases in the number of PNA + cells and ONL thickness and cell layers in ES-treated 9-weeks-old *Rho* ^*−/−*^ mice compared to those with sham stimulation (Figures 6C–E; *P* < 0.001). These results highlight the efficiency of TpES in preserving outer segment morphology and improving cone survival.

## Discussion

Our research demonstrated that treatment of TpES using a ramp waveform, especially when given every other day, represents an effective non-invasive procedure for preserving photoreceptor morphology, survival, and function in an RP model. In *Rho* ^*−/−*^ mice, photoreceptor degeneration begins early in life; by 6 weeks of age, they can lose over 40% of photoreceptors (Supplementary Figure S2) while still maintaining a normal VA of ∼0.45 cycle/degree, comparable to that seen in adult C57BL/6J wild-type mice. However, by 9 weeks of age, the VA in *Rho* ^*−/−*^ mice decreases to ∼0.34 cycle/degree and continues to decline until complete blindness occurs by about 4 months of age (Xiao et al., 2019). Our present studies showed that TpES, particularly at the ramp waveform, maintained the VA in *Rho* ^*−/−*^ mice nearly unchanged from 6 to 9 weeks of age. The ramp waveform was more effective than the commonly used rectangular waveform in promoting photoreceptor survival, and the therapeutic benefits of unilateral ramp-TpES extended to the contralateral eye in *Rho* ^*−/−*^ mice. Defining and standardizing the parameters of ES are critical for clinical practice applications.

Currently, rectangular waveforms are commonly applied to preclinical and clinical ES investigations (Sehic et al., 2016). We showed that a ramp waveform propagates the electrical field more efficiently in the mouse and human cadaver eyes, generating 30 times less conductive resistance than the rectangular waveform (Enayati et al., 2024). This suggests that ES at a ramp waveform elicits biological effects at a much higher efficiency or with a lower current amplitude. In the present study, we noted that TpES administered unilaterally at a ramp waveform not only improved VA, CS, and photopic ERG b-wave amplitudes in the treated eye, but its therapeutic benefits spread to the contralateral eye. In contrast, the benefit of rectangular TpES at the same current intensity was limited to the treated eye, resulting in improvement in VA, but not CS values These observations are in line with our previous reports (Yu et al., 2020; Enayati et al., 2024), supporting that the ramp waveform is more efficient in delivering electrical field than the rectangular waveform. Thus, the ramp waveform is advantageous in enhancing the survival and functionality of retinal neurons with a wider safety profile by requiring lower electrical intensity and featuring lower impedance than rectangular ES waveforms in delivering an electrical field to the posterior eye. In agreement with this finding, the ramp waveform at a similar amplitude to the rectangular waveform required far less voltage potential to reach the current amplitude needed for stimulating responses in cochlear neurons (Navntoft et al., 2020). This explains the significantly decreased neuroprotective impact of rectangular ES compared to ramp ES when it is delivered at the same current amplitude in *Rho* ^*−/−*^ mice.

Other clinical studies showed that regular use of trans-corneal ES also decreased the loss of visual field area in individuals with RP compared to untreated eyes in a dose-dependent manner; trans-corneal ES treatment was most effective when was delivered at above 0.8–1.0 mA (5 ms/phase, 20 Hz) (Stett et al., 2023). It should be noted that an increase in current intensity is not linearly correlated with the neuroprotective effect elicited by ES, and higher ES amplitude may become detrimental to neuronal cells. Morimoto et al. reported that significant improvement in neuron survival in the retinas of adult rats was seen when the ES was raised to 100 μA and 200 μA. However, the mean retinal ganglion cell densities dropped to 70.0% and 64.5%, respectively, when ES amplitude was increased to 300 μA and 500 μA (Morimoto et al., 2010). Our data suggest that ES at a ramp waveform may present a better safety profile due to its requirement of much lower current intensity to penetrate the eye tissues.

It is encouraging to note that TpES at the current parameters did not cause tissue heating, skin burns or any other damage in anesthetized mice after 4-min biphasic ES *in vivo* (Enayati et al., 2024). An *in vitro* study reported that ES of biphasic waveforms at amplitudes over 500 µA induced cell toxicity (Lennikov et al., 2022). These data are consistent with clinical observations (Rizzo et al., 2003; Cogan et al., 2016). Maintaining the structural integrity of retinal cellular components is directly linked to the protective functions of TpES. Our previous studies further demonstrated that transpablpebral ES (TpES) represents a safer and more effective ES approach for treating retinal neurodegeneration clinically without disrupting corneal mucin homeostasis or causing corneal epithelial damage compared to trans-corneal ES (Yang et al., 2022).

In summary, we demonstrated that TpES increased the ONL thickness, the density of cones and rods, and the morphology of the inner and outer segments. The data suggest that ES enhances cell survival and prevents photoreceptor degeneration. Our research further indicates that these benefits are sustainable with continued ES application. Our findings provide valuable insights for future study and possible therapeutic uses of noninvasive ES for photoreceptor degenerative diseases.

## Statement of limitations

1. As we used 6-week-old *Rho* ^*−/−*^ mice in the present studies, mice have already lost nearly 40% photoreceptors. We do not yet know if earlier starts of TpES could have generated better functional and morphological outcomes.
2. The mice in longer-term studies of TpES every day, every other day, and every 3-day treatment groups were not controlled for the number of anesthetic events. In our study, the everyday stimulation group and the sham control group received twice as many isoflurane anesthetic events as the every-other-day stimulation group. We do not know if a higher number of anesthetic events in the daily TpES group of mice may have the potential to decrease animal performance in behavioral studies such as OMR, resulting in masked or diminished VA and CS values due to elevated stress.

## Data Availability

The original contributions presented in the study are included in the article/supplementary material, further inquiries can be directed to the corresponding author/s.
