# Supplementary material for: Preservation of vision by transpalpebral electrical stimulation in mice with inherited retinal degeneration
Source: Front Cell Dev Biol. 2024 Aug 14;12:1412909. doi: 10.3389/fcell.2024.1412909 (PMC11349514; doi:10.3389/fcell.2024.1412909)
Supplement: Supplementary file 1 [file DataSheet1.docx]

Supplementary Material

Preservation of Vision Function by Transpalpebral Electrical Stimulation in Mice with Inherited Retinal Degeneration

Kasim Gunes^1, 2^, Karen Chang^1^, Anton Lennikov^1^, Wai Lydia Tai^1^, Julie Chen^1^, Farris ElZaridi^1^, Kin-Sang Cho^1^, Tor P. Utheim^1, 3, 4^, Dong Feng Chen^1^*

^1^1Schepens Eye Research Institute of Mass Eye and Ear, Department of Ophthalmology, Harvard Medical School, Boston, MA, United States.
^2^Department of Histology and Embryology, School of Medicine, Marmara University, Türkiye ^3^Department of Medical Biochemistry, Oslo University Hospital, Norway
^4^Department of Ophthalmology, Oslo University Hospital, Norway

*** Correspondence:**

Dong Feng Chen

[dongfeng_chen@meei.harvard.edu](mailto:dongfeng_chen@meei.harvard.edu)

## Supplementary Figures:
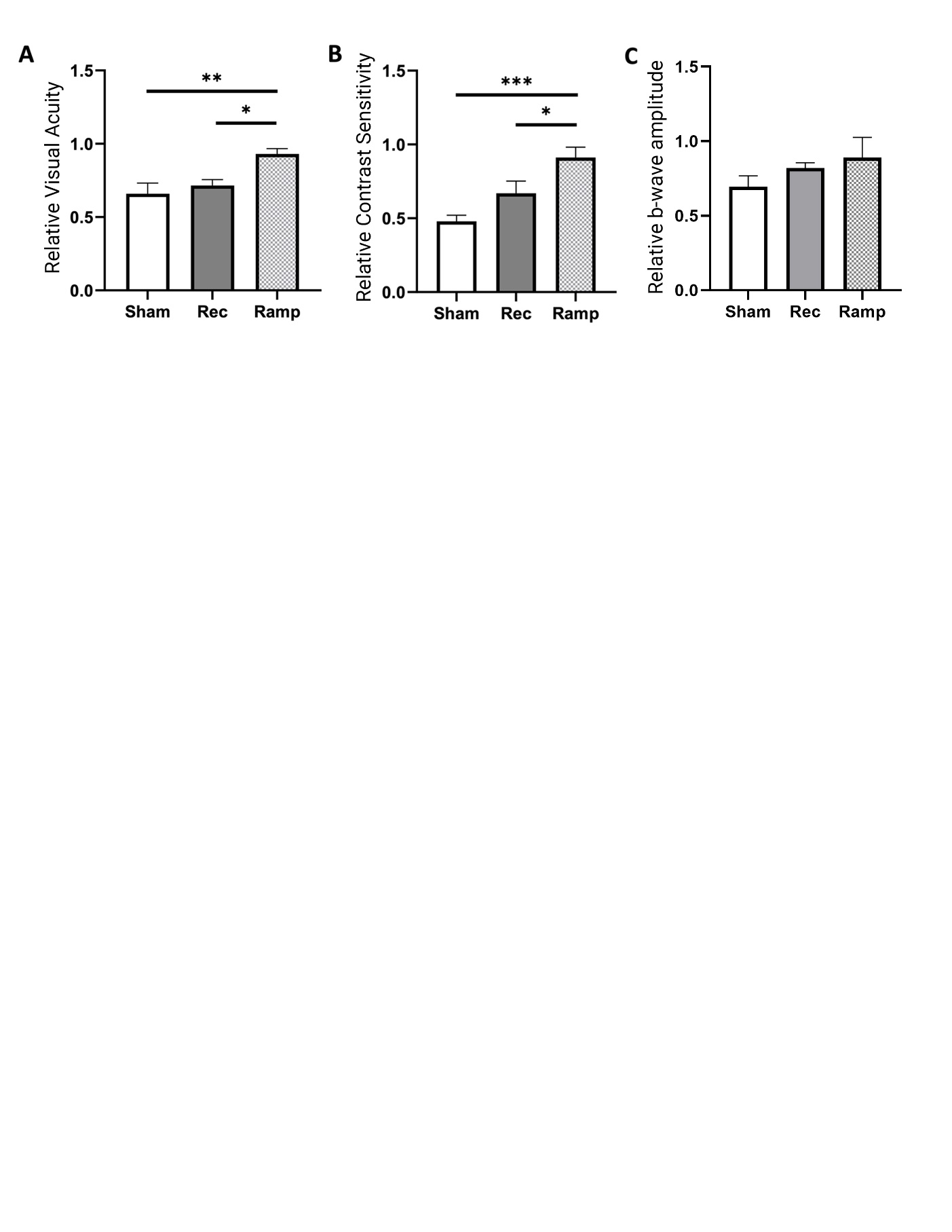


**Figure S1. Ramp, but not rectangular-ES, improves eyesight in *Rho*^−/−^ mice in the eye contralateral to the treatment.** VA (A), CS (B), and b-wave amplitude of photopic ERG (C) taken from the sham-treated eye or eyes contralateral to the rectangular (Rec) or ramp (Ramp) stimulations. Data are presented as VA or CS values relative to their baseline levels acquired before the initial stimulation at 6 weeks and established as 1. Photopic 600 ERG recordings from *Rho^-/-^* mice showing quantification of b-wave amplitudes and (E) representative ERG plot taken 7 days of ES or Sham treatment (F). Data are presented as values relative to their baseline levels acquired before the initial stimulation at 6 weeks and established as 1. Statistical significance was evaluated using one-way analysis of variance (ANOVA), with p-values < 0.05 deemed significant. For all statistical data, an asterisk indicates * p < 0.05, ** p < 0.01, and values are reported as mean ± SEM. (VA, CS n = 7 mice/group; ERG n = 6 mice/group)


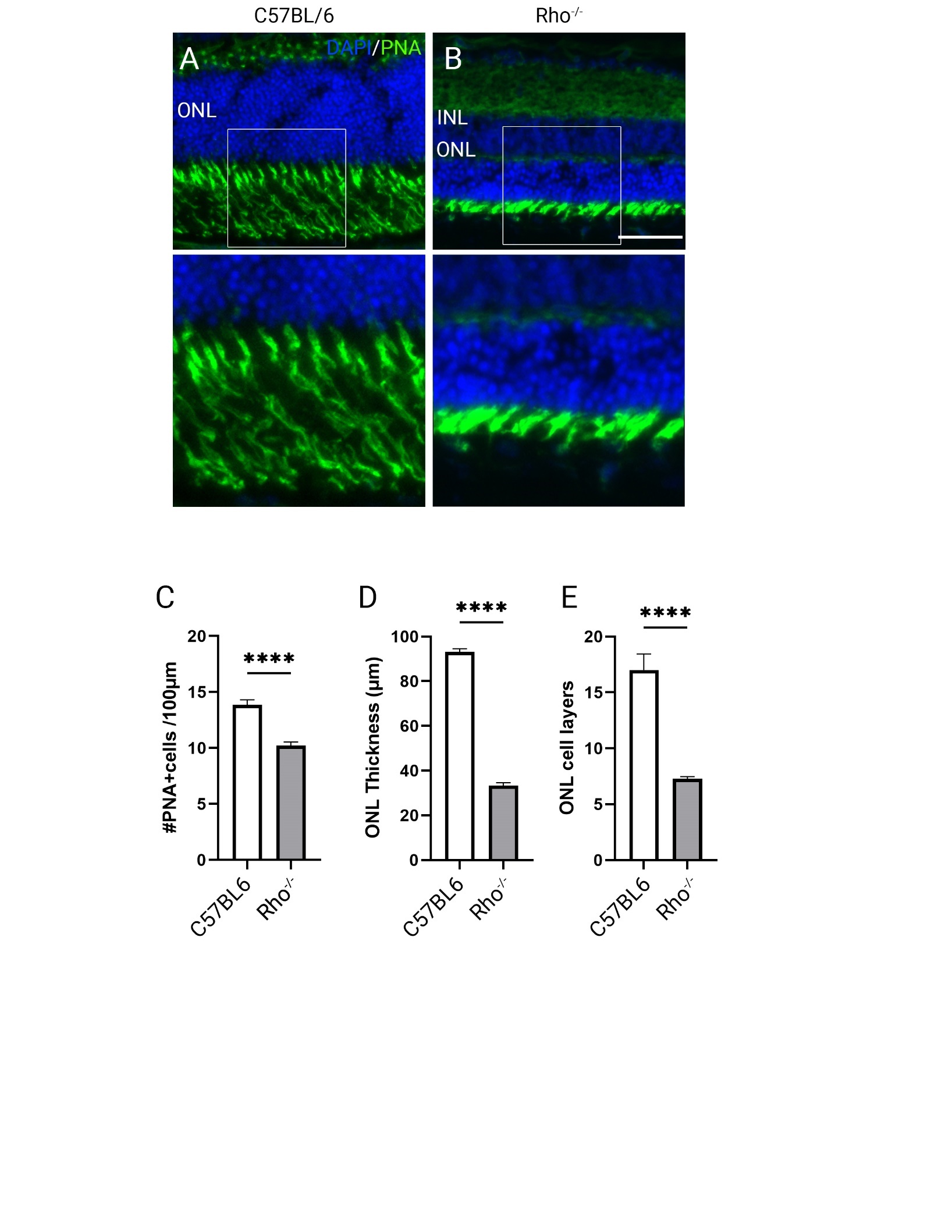


**Figure S2. Progressive photoreceptor degeneration in *Rho*^−/−^ mice.** Representative morphologies of retinal sections immunolabeled with Peanut agglutinin (PNA) and counter-stained with 4’,6-diamidino-2-phenylindole (DAPI) from 6-weeks-old C57BL/6 (WT) (A), 6-weeks-old Rho-/- mice (B). ONL: outer nuclear layer; INL: inner nuclear layer. Scale bar = 50 μm; insert =20 μm. Quantification of PNA-positive cells across retinal sections (E). Measurement of retinal ONL thickness (F) and ONL cell layer qualifications of the retinal sections (G). Statistical significance was evaluated using one-way ANOVA. *P < 0.05, **P < 0.01 **** P < 0.001, and values reported as mean ± SEM (C5BL6 n = 6 mice/group; Rho-/- mice n = 8 mice/group).

##
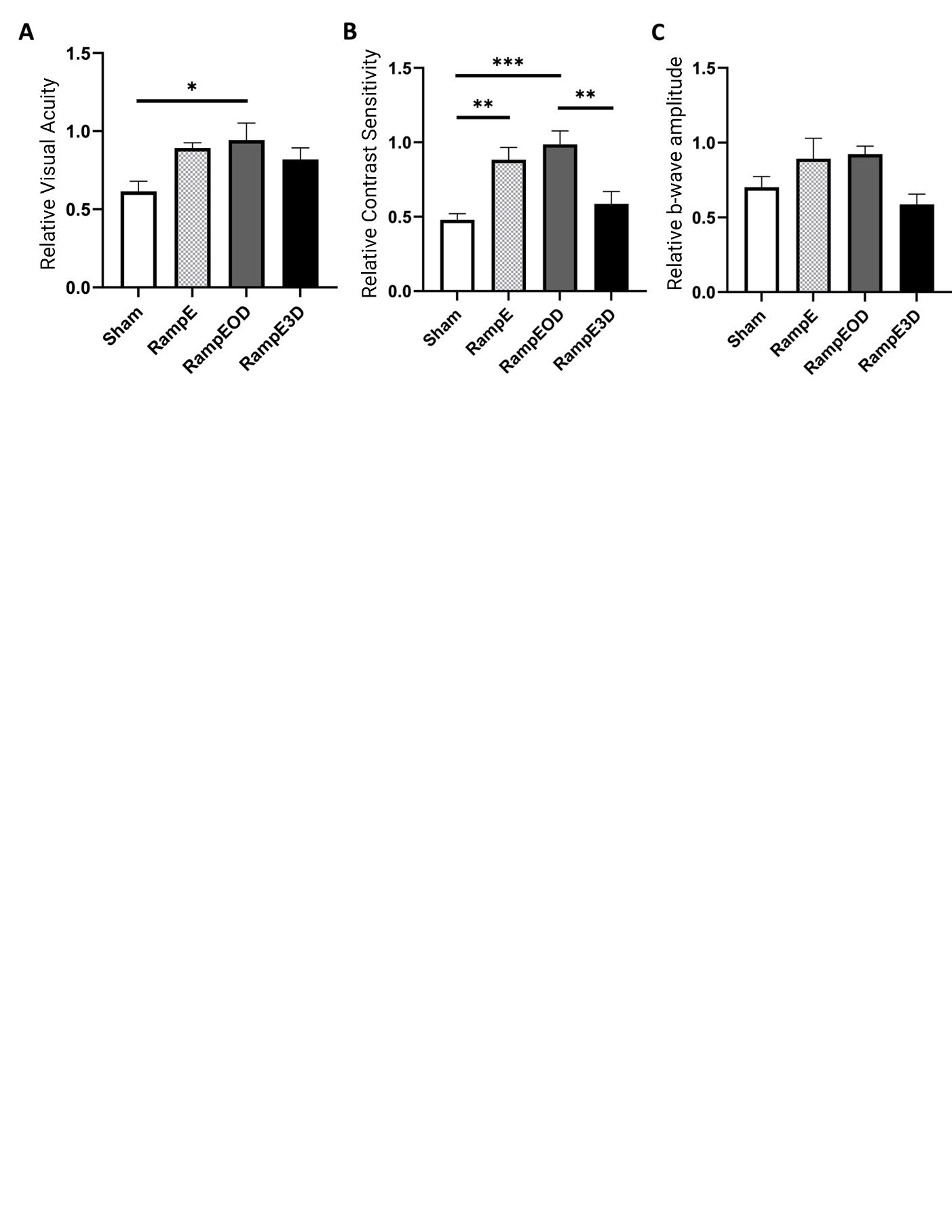


**Figure S3. Ramp, but not rectangular-ES, improves eyesight in *Rho*^−/−^ mice in the eye contralateral to the treatment.** VA (A), CS (B), and b-wave amplitude of photopic ERG (C) taken from the sham-treated eye or eyes contralateral to the rectangular (Rec) or ramp (Ramp) stimulations. Data are presented as VA or CS or ERG values relative to their baseline levels acquired before the initial stimulation at 6 weeks and established as 1. Statistical significance was evaluated using one-way analysis of variance (ANOVA), with p-values < 0.05 deemed significant. For all statistical data, an asterisk indicates * p < 0.05, ** p < 0.01, and values are reported as mean ± SEM. (VA, CS n = 7 mice/group; ERG n = 6 mice/group)


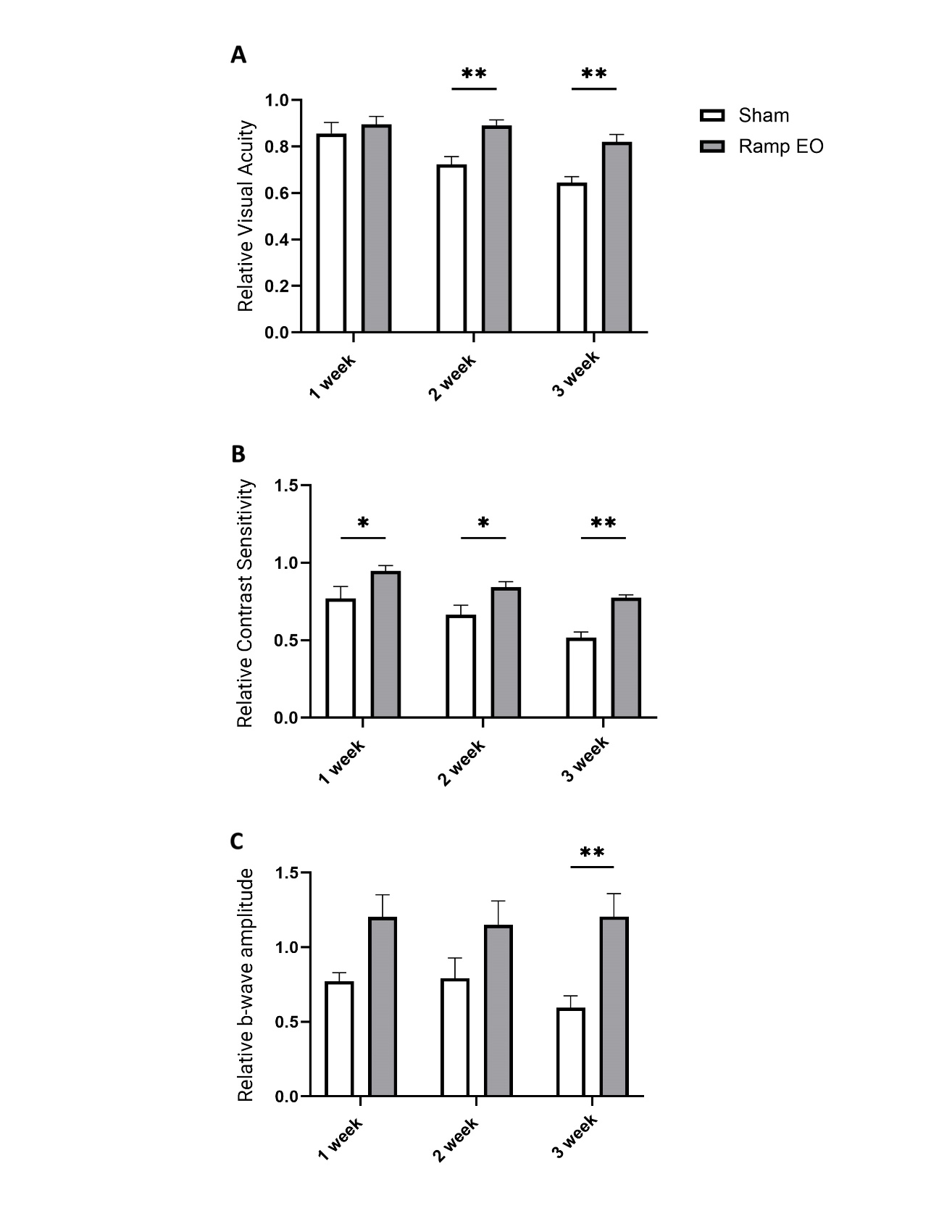


**Figure S4. TpES with a ramp waveform prevents vision loss in *Rho*^−/−^ mice in eyes contralateral to the ES treatment.**  VA (A), CS (B), and b-wave amplitude of photoreceptor ERG (C) taken from the sham-treated eyes or eyes contralateral to ES treatment in *Rho*^−/−^ mice over a 3-week period. Data are presented as VA or CS or ERG values relative to their baseline levels acquired before the initial stimulation at 6 weeks and established as 1. The statistical method used was the Holm-Šídák test with multiple comparisons corrections. For all statistical data, an asterisk indicates *p < 0.05, **p < 0.01, and values are reported as mean ± SEM (n = 8 mice/group).
